# Case Report: Newly discovered ELN gene mutation in congenital heart disease: case analysis and review

**DOI:** 10.3389/fped.2026.1671066

**Published:** 2026-01-29

**Authors:** Peiwen Cheng, Guozhen Wang, Jialin Qiu, Xia Xie, Yong An

**Affiliations:** 1 National Clinical Research Center for Child Health and Disorders, Ministry of Education Key Laboratory of Child Development and Disorders, Chongqing Key Laboratory of Structural Birth Defect and Reconstruction, Department of Thoracic Surgery, Children’s Hospital of Chongqing Medical University, Chongqing, China; 2 Department of Respiratory Medicine, Children’s Hospital of Chongqing Medical University, Chongqing, China

**Keywords:** case report, elastin mutation, frameshift variant, haploinsufficiency, pediatric cardiology, supravalvular aortic stenosis

## Abstract

**Introduction:**

Supravalvular aortic stenosis (SVAS) is a rare left ventricular outflow tract obstruction, most commonly caused by pathogenic variants in ELN. Truncating variants in exons 1–29 typically produce non-syndromic SVAS through elastin haploinsufficiency, whereas C-terminal variants are linked to autosomal dominant cutis laxa. However, clinically and mechanistically well-characterized variants in the distal part of this “stenotic zone,” such as exon 28, remain uncommon.

**Methods:**

We conducted a retrospective family-based case report with standardized clinical evaluation, serial echocardiography, and trio whole-exome sequencing with Sanger confirmation and conservation analysis.

**Results:**

A female infant presented at 1 month with severe sinotubular junction narrowing (Z-score −4.8, peak gradient 24 mmHg), severe peripheral pulmonary artery stenosis, a small atrial septal defect, and moderate mitral regurgitation. Her father had severe SVAS with mild PPAS and prior aortic root enlargement, without syndromic features. Trio sequencing identified a novel heterozygous ELN exon 28 frameshift variant, c.1879_1883dup (p.Ala629LeufsTer15), inherited from the father. Ala629 is fully conserved, and the duplication introduces a premature stop codon, consistent with nonsense-mediated decay and elastin haploinsufficiency. At 9 months, SVAS progressed (peak gradient 35 mmHg), while PPAS gradients regressed by >40%.

**Conclusion:**

This novel exon 28 ELN frameshift expands the non-syndromic SVAS spectrum and illustrates a characteristic pattern of progressive aortic stenosis with improving PPAS, supporting ELN testing and targeted longitudinal surveillance in similar patients and families.

## 1 Introduction

Congenital heart disease (CHD) is one of the most common birth defects in newborns, with a global incidence of approximately 8–12 per 1,000 live births; severe forms requiring intervention occur in about 6 per 1,000 (1). In recent years, advances in prenatal screening and ultrasonographic diagnosis have led to a rising reported prevalence of CHD, especially in Asia where it now exceeds that in Europe and North America, possibly reflecting genetic susceptibility or environmental factors (2). CHD remains the leading cause of infant mortality from non-infectious causes. However, surgical and catheter-based interventions have significantly improved survival, resulting in a growing population of adult CHD patients. Genetic factors play a crucial role in CHD pathogenesis: whole-exome sequencing studies indicate that approximately 10%–35% of CHD cases can be attributed to single-gene variants, copy number variations, or chromosomal abnormalities, with *de novo* and rare inherited variants contributing significantly (3).

Severe supravalvular aortic stenosis (SVAS) is a rare obstructive lesion of the left ventricular outflow tract, with an incidence of roughly 1 in 20,000–25,000 live births, accounting for 8%–14% of all forms of congenital aortic stenosis (4, 5). SVAS typically manifests as a localized or diffuse narrowing at the sinotubular junction or ascending aorta, leading to increased left ventricular afterload, left ventricular hypertrophy, and systemic hypertension. Clinically, a heart murmur is often the first finding; in severe cases, affected infants or children may develop angina, syncope, or heart failure. SVAS is frequently accompanied by peripheral pulmonary artery stenosis (PPAS), especially in infancy, which can cause right ventricular pressure overload. This condition can occur in isolation (non-syndromic SVAS) or as part of Williams–Beuren syndrome (WBS), and it is inherited in an autosomal dominant manner with highly variable expressivity and penetrance (4).

The elastin gene (ELN) is located on chromosome 7q11.23, spans approximately 45 kb, and comprises 34 exons (exons 34–35 are absent in primates) (6). It encodes tropoelastin, a soluble precursor protein that is cross-linked by lysyl oxidase to form insoluble elastic fibers. Elastin is a major component of the arterial wall and the medial elastic laminae, conferring elasticity and recoil to large arteries to maintain laminar blood flow and buffer pulse pressure (7). ELN expression is tightly regulated in a spatiotemporal manner: it peaks in late embryonic and early postnatal stages and is markedly downregulated in adulthood, influenced by various transcription factors (e.g., TGF-β, IGF-1, glucocorticoids) and microRNAs (e.g., the miR-29 and miR-15 families). Post-transcriptional regulation—including mRNA stability and post-translational modifications—is also critical for elastin homeostasis. Notably, in adult vasculature, elastin has an extraordinarily long half-life (on the order of decades) with virtually no turnover (6, 8).

Diseases caused by ELN variants, collectively termed elastin arteriopathy, can be categorized into three groups based on the variant type and pathogenic mechanism (Table 1). Truncating variants (nonsense, frameshift, or splice-site mutations affecting exons 1–29) trigger nonsense-mediated mRNA decay and result in functional elastin haploinsufficiency; clinically, these cause non-syndromic (familial) SVAS often accompanied by infantile PPAS that typically improves spontaneously (7, 11). In contrast, C-terminal variants (missense or in-frame mutations in exons 30–34) produce an abnormal tropoelastin with a dominant-negative effect on elastic fiber assembly, primarily manifesting as autosomal dominant cutis laxa (ADCL) with relatively mild vascular involvement (13). Finally, a large 7q11.23 microdeletion encompassing ELN and 25–28 neighboring genes gives rise to WBS, a complex multisystem disorder characterized by SVAS, distinctive facial features, intellectual disability, and hypercalcemia (9, 35).

**Table 1:** Molecular mechanisms and clinical phenotypic classification of ELN-related diseases.

Molecular lesion type	Typical variant type	Primary mechanism	Typical clinical phenotype	Incidence of peripheral pulmonary artery stenosis (PPAS)	Prognostic features and intervention frequency	References
7q11.23 microdeletion	Involving ELN and ≥25–28 adjacent genes (typical 1.4–1.8 Mb deletion)	Multigene hemizygosity	Williams-Beuren syndrome (SVAS + characteristic facies, intellectual disability, hypercalcemia, growth retardation)	High incidence (>70%), significant in infancy, partial improvement with age but high persistence	Multisystem involvement, high intervention frequency, restenosis common	(4, 9, 10)
Truncating/Splice variants (exons 1–29)	Nonsense, frameshift, splice site variants (>100 reported)	NMD → functional haploinsufficiency	Nonsyndromic/familial SVAS ± peripheral PPAS, no extracardiac syndrome features	50%–75% of children present in infancy, often naturally improve with age (>50% cases)	Primarily vascular phenotype, early intervention but lower reintervention rate	(5, 7, 11, 12)
C-terminal missense/in-frame variants (exons 30–34)	Missense, in-frame insertions/deletions	Dominant-negative protein interferes with assembly	Autosomal dominant cutis laxa (ADCL) ± mild vascular disease (e.g., mild SVAS)	Rare (<10%)	Primarily skin, mild vascular, better prognosis	(8, 13)

Among non-syndromic SVAS patients with truncating ELN variants, PPAS is observed in infancy in approximately 50%–75% of cases. It often presents as bilateral, multifocal branch pulmonary artery stenoses that lead to right ventricular hypertension. However, unlike in WBS, PPAS in the non-syndromic context generally has a benign natural history: as the child grows, the pulmonary arteries often undergo “catch-up” growth, and the stenoses markedly lessen or even resolve, usually without the need for intervention (10, 12). This phenomenon may be related to the higher requirement for elastin and the greater capacity for vascular remodeling in infancy. Moreover, large cohort studies have confirmed that the spontaneous improvement of PPAS is an important clinical clue distinguishing non-syndromic SVAS from syndromic (WBS) cases (14). In this study, we report a case involving a novel heterozygous frameshift variant in ELN exon 28 (c.1879_1883dup) that causes a typical truncating effect, thereby expanding the mutational spectrum of ELN.

## 2 Patients and methods

This study was a retrospective case report of a single family, approved by the Ethics Committee of the Children's Hospital of Chongqing Medical University (Approval No. 2025-434). The parents of the proband both signed written informed consent, agreeing to clinical evaluation, genetic testing, and use of anonymized data for research publication, in accordance with the 2013 version of the Declaration of Helsinki (15).

### 2.1 Clinical evaluation

The child underwent a standardized pediatric cardiology evaluation, including detailed medical history, physical examination (with emphasis on excluding the typical extra-cardiac features of Williams-Beuren syndrome, such as distinctive facial features, loose skin, growth retardation, hypercalcemia, neurocognitive abnormalities, etc.), and cardiac imaging examinations. Transthoracic echocardiography was performed by a physician with at least 10 years of experience in accordance with the American Society of Echocardiography (ASE) pediatric guidelines (16). Cardiac CT angiography (CTA) or magnetic resonance imaging (MRI) of the great vessels was only considered for surgical planning or if additional vascular involvement was suspected; in this study, echocardiography was prioritized as the diagnostic and follow-up tool.

### 2.2 Genetic analysis

Peripheral blood (2 mL) was collected from the patient and parents for genomic DNA extraction, followed by trio whole-exome sequencing. Suspected pathogenic variants were confirmed by bidirectional Sanger sequencing. The reference sequence for ELN was NM_000501.4 (protein NP_000492.3), and variant nomenclature followed the HGVS guidelines. Pathogenicity of variants was evaluated according to the American College of Medical Genetics and Genomics (ACMG) and Association for Molecular Pathology (AMP) guidelines for variant interpretation (17). Additionally, a multi-species sequence alignment of the elastin protein (encoded by ELN) was performed to assess evolutionary conservation.

### 2.3 Literature and database search

A systematic search of PubMed was conducted using the keywords: (“*supravalvular aortic stenosis*” *OR* “*SVAS*” *OR* “*elastin arteriopathy*”) *AND* (“*ELN*” *OR* “*elastin*”) *AND (frameshift OR nonsense OR truncating)*. The references of the identified studies were also manually reviewed to avoid omissions. In addition, the ClinVar database (NCBI) and the Human Gene Mutation Database (HGMD) were searched for all reported ELN variants, focusing on those classified as “Pathogenic/Likely pathogenic” with the phenotype “Supravalvular aortic stenosis, autosomal dominant”. After removing duplicates, variants were included if they met all of the following criteria: 1. Clear HGVS nomenclature is provided. 2. Detailed clinical phenotype is described [including whether accompanied by peripheral pulmonary artery stenosis (PPAS) or extra-cardiac features]. 3. Supporting functional validation evidence is available.

Finally, more than 140 pathogenic ELN variants were included for constructing the mutation spectrum in Figure 4 and for classification in Supplementary Appendix 1.

## 3 Results

**Figure 1:**
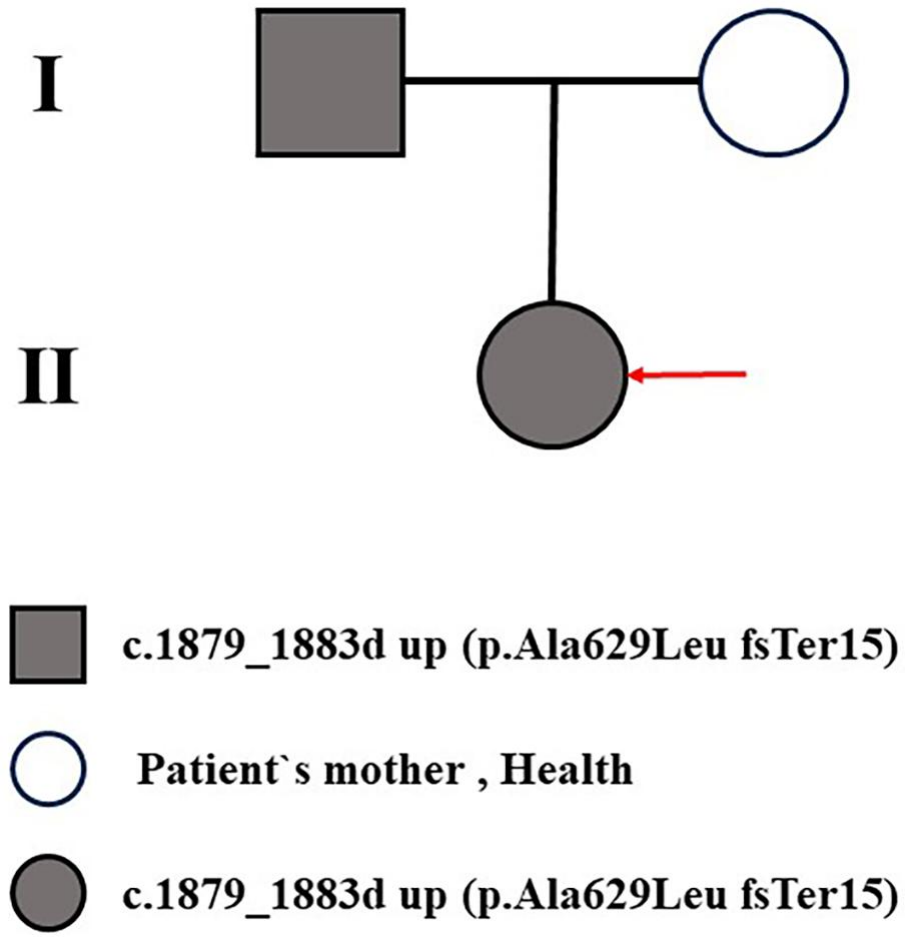
Family pedigree consists of one proband. I-1 represents the proband's father; I-2 represents the proband's mother; II-1 represents the proband (indicated by an arrow).

The proband was referred after a grade III/6 systolic murmur was detected on routine neonatal examination. At presentation, her weight was 3.5 kg and length 50 cm, with normal growth and development. Physical examination did not reveal any extra-cardiac features suggestive of Williams–Beuren syndrome. Initial transthoracic echocardiography at 1 month of age demonstrated severe narrowing at the sinotubular junction (inner diameter 6.7 mm, *Z*-score −4.8, peak systolic gradient 24 mmHg), severe stenosis of the main and branch pulmonary arteries (right ventricle–pulmonary artery gradient 55 mmHg; left branch 58 mmHg; right branch 65 mmHg), a small atrial septal defect (4 mm, left-to-right shunt), and moderate mitral regurgitation.The proband's father had been diagnosed in adulthood with severe supravalvular aortic stenosis (SVAS) and mild peripheral pulmonary artery stenosis, for which he had undergone aortic root enlargement surgery with good postoperative recovery and no syndromic features. Given this family history, a hereditary etiology was strongly suspected (Figure 1).

**Figure 2:**
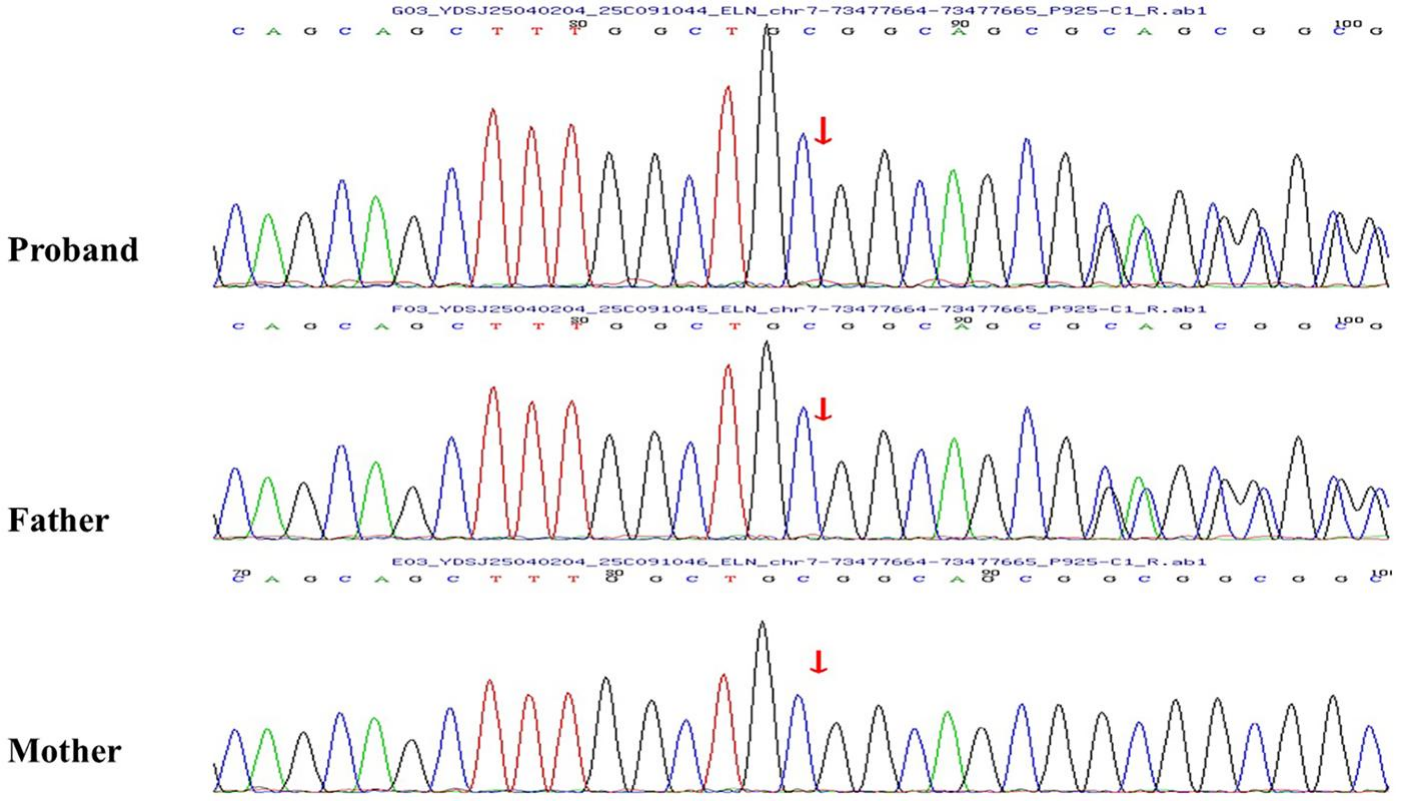
The *ELN* mutation site. The red arrow indicates the site of the heterozygous 5-bp duplication (c.1879_1883dupGGCCC).

Peripheral blood genomic DNA was therefore obtained from the proband and both parents for trio whole-exome sequencing (Trio-WES) and Sanger confirmation. This identified a novel heterozygous frameshift variant in ELN in the proband and her father: NM_000501.4 (exon 28): c.1879_1883dup (p.Ala629LeufsTer15) (Figure 2). The mother carried the wild-type sequence, confirming paternal inheritance. To date, this variant has not been reported in ClinVar, HGMD, or PubMed and is therefore considered a previously unreported pathogenic variant.A typical 5-bp repeat with superimposed peaks originating at the duplicated motif (red arrows) was observed in both the proband and her father, which is characteristic of a heterozygous repeat insertion, whereas the mother's chromatogram was normal. These findings provided definitive evidence for the heterozygous state of the variant and its strictly paternal inheritance.

**Figure 3:**
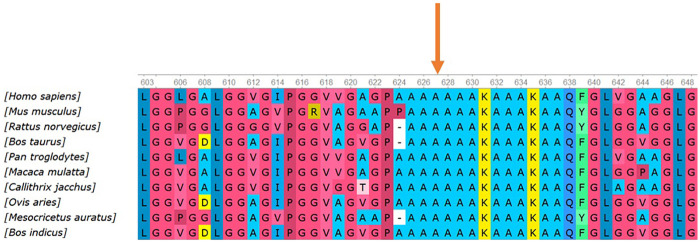
Conservation analysis of the ELN gene. The orange arrow highlights alanine 629 (p.Ala629), which is 100% conserved across 12 vertebrate species (Homo sapiens, Pan troglodytes, Mus musculus, Rattus norvegicus, Bos Taurus, Ovis aries, Canis lupus familiaris, Gallus gallus, etc.).

**Figure 4:**
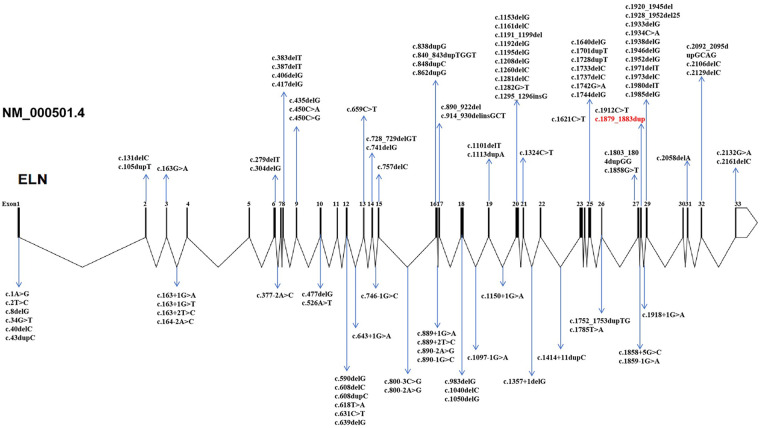
Elastin gene structure and pathogenic variants to date. Known exonic and intronic variants curated from published case reports, ClinVar and HGMD. The variant identified in this study (c.1879_1883dup) is highlighted in red.

Multi-species conservation analysis further supported its pathogenicity (Figure 3). Alanine at position 629 (p.Ala629) lies within a highly conserved functional domain of the ELN protein and is completely identical across 12 species, including human, chimpanzee, mouse, rat, cow, sheep, dog, chicken, and zebrafish (100% conservation). The affected alanine residue (p.Ala629) is indicated by an orange arrow. The 5-bp duplication at this site results in a completely disrupted downstream amino acid sequence and introduces a premature termination codon 15 codons downstream (Ter15), which is predicted to trigger nonsense-mediated mRNA decay (NMD) and cause functional elastin haploinsufficiency. To define the position of this variant within the ELN mutational spectrum, we integrated previously reported pathogenic variants (Figure 4; details are provided in the Appendix). The schematic representation of the ELN gene shows 34 exons, with truncating variants densely clustered between exons 10 and 30.

Given the proband's initial mild SVAS, a conservative management approach was adopted with close follow-up. At 9 months of age, the child's weight had increased to 8 kg with normal growth and no new symptoms, but serial echocardiography revealed important hemodynamic changes. The supravalvular aortic stenosis progressed: the sinotubular junction (aortic root) diameter narrowed from 6.7 mm at presentation to 5.6 mm, corresponding to a *Z*-score of −6.2, and the peak systolic pressure gradient across the lesion rose to about 35 mmHg (up from 24 mmHg initially). In contrast, the peripheral pulmonary artery stenoses showed significant spontaneous improvement—the peak gradient in the left pulmonary artery dropped to 29 mmHg (from 58 mmHg) and in the right to 50 mmHg (from 65 mmHg), a >40% reduction without any intervention. Furthermore, an atrial septal defect that had been present closed spontaneously over this time, and a previously moderate mitral regurgitation diminished to only mild. These dynamic trends mirror observations in larger cohorts of non-syndromic elastin arteriopathy. In particular, SVAS tends to worsen progressively during infancy, often requiring surgical or catheter intervention in the first years of life (the median age at first intervention is 1.1 years in one study of ELN mutation carriers) (14).

## 4 Discussion

### 4.1 Mutation types and distribution: Non-syndromic ELN gene mutational Spectrum

To date, more than 140 pathogenic or likely pathogenic variants of the ELN gene have been reported in the literature, with non-syndromic cases accounting for about 60% (6). Non-syndromic ELN mutations are highly diverse in type, including frameshift mutations (small insertions/deletions causing reading frame shifts), nonsense mutations (introducing a premature termination codon, PTC), splice-site mutations (affecting normal mRNA splicing and leading to exon skipping or aberrant insertions), and a small number of missense mutations (single amino acid substitutions) (7, 11, 13). The vast majority of these mutations are heterozygous. The core pathogenic mechanism is loss-of-function (LOF), i.e., the heterozygous variant causes elastin functional haploinsufficiency, with approximately a 50% reduction in elastin production (8). According to the latest review in 2025, LOF variants account for over 85% of mutations in SVAS patients, with frameshift and nonsense mutations comprising about two-thirds of these (11). A representative multi-center Chinese study (Zhou et al.) found that 9 out of 11 pathogenic variants were truncating (5 nonsense + 4 frameshift) and only 2 were missense, further confirming the dominance of LOF variants (5). A new report likewise confirmed that a novel ELN variant caused isolated SVAS via an LOF mechanism. Missense mutations are relatively rare (<10%) and usually occur only at the *C*-terminus of the protein, where they can exert a dominant-negative effect. Splice-site variants can indirectly result in frameshifts or deletions (for example, by activating cryptic splice sites), and small in-frame exon deletions or internal microduplications can also lead to loss of elastin function (17). Overall, LOF-type variants constitute the vast majority of non-syndromic ELN mutations, whereas missense mutations are mostly associated with autosomal dominant cutis laxa (ADCL).

Mutation distribution within the ELN gene shows a striking regional difference—one of the most fascinating features of ELN mutations. Virtually all mutations associated with SVAS and other arterial stenoses are located in the first 29 exons. Variants in this region typically trigger efficient nonsense-mediated mRNA decay (NMD), leading to “pure” haploinsufficiency (18). The resultant 50% reduction in elastin protein impairs elastic fiber formation in the arterial wall, causing medial layer underdevelopment and luminal narrowing. By contrast, ADCL-related mutations are highly enriched in exons 30–34 (the 3′ end of the gene) and are often missense or late frameshift changes. These lead to translation read-through beyond the normal stop codon and production of an abnormally extended elastin protein that escapes NMD. The mutant elastin incorporates into elastic fibers and disrupts their assembly via a dominant-negative mechanism, which is the main cause of cutis laxa, aortic root dilatation, and emphysema observed in ADCL patients (9, 19).

This general rule “mutations in the first 29 exons tend to cause arterial stenosis phenotypes, whereas mutations in the terminal exons produce an abnormal protein leading to cutis laxa and arterial dilatation phenotypes” has been validated by multiple independent cohorts. For example, it was observed in the European cohort of Metcalfe et al. (7), an international multi-center study by Min et al. (14), and the Chinese cohort reported by Zhou et al. (5). The “position effect” hypothesis proposed by Duque Lasio et al. (8) also reflects this mechanism, and a review by Ganjibakhsh et al. (20) noted no major exceptions to this pattern. However, there are occasional exceptions—for instance, one study reported that a missense mutation in exon 25 can also lead to ADCL. Thus, although the phenotypes of non-syndromic ELN mutations generally follow the above rule, the diversity of individual mutations and phenotypes reminds us to consider potential variability during diagnosis.

### 4.2 Clinical phenotype: cardiovascular malformations caused by ELN mutations

The primary congenital cardiovascular anomaly caused by ELN mutations is localized stenosis of large arteries, with SVAS being the most characteristic feature. SVAS can occur in isolation, but it often coexists with other vascular stenoses—especially pulmonary artery stenosis (PAS), among which peripheral pulmonary artery stenosis (PPAS) is the most common (14). Elastin haploinsufficiency leads to underdevelopment of large elastic arteries: the arterial walls become abnormally thick and less elastic, predisposing to segmental narrowing. In this context, supravalvular aortic narrowing is the most frequent lesion, and secondary pulmonary artery stenosis is often present concomitantly (4, 21, 22). Statistics indicate that about 30% of patients with non-syndromic ELN mutations will require surgical intervention for the stenotic vessels (23).

Aside from the aorta and pulmonary artery, other peripheral elastic arteries may also be affected (24). Some cases exhibit segmental narrowing of the superficial temporal arteries and other medium-sized arteries, and there may even be involvement of intracranial arteries in the anterior circulation—presenting as arterial stenoses or aneurysm formation. In addition, coronary artery stenosis or occlusion is common in severe SVAS, which can lead to myocardial ischemia or even sudden death; therefore, vigilant monitoring of the coronary arteries is required in these patients (25, 26). Notably, whereas elastin haploinsufficiency (as seen in isolated ELN mutations) usually does *not* cause skin laxity or other systemic connective tissue symptoms, Williams-Beuren syndrome (WBS) patients *do* typically have soft, loose skin and multi-system abnormalities. This difference underscores the phenotypic contrast between isolated ELN haploinsufficiency and the contiguous gene deletion of WBS (27).

It is worth mentioning that some carriers of ELN mutations may exhibit no obvious symptoms—a phenomenon known as incomplete penetrance. For example, in certain families, members carrying the same pathogenic variant might only have mild arterial narrowing detectable by imaging (with no clinical symptoms), and some carriers may be entirely normal (4, 5, 22). This incomplete penetrance is not uncommon in ELN mutations, suggesting that, beyond the mutation itself, there are likely modifier genes or environmental factors that influence the severity of the phenotype (27).

In non-syndromic ELN-mutation SVAS patients, apart from arterial lesions, a subset of patients present with extracardiac features such as inguinal hernia or diaphragmatic hernia (28–30). These manifestations may relate to connective tissue abnormalities, indicating that ELN mutations can lead to other connective tissue defects beyond the vasculature (29, 31, 32). In contrast to ADCL patients—who typically show aortic root dilatation, pulmonary artery enlargement, and cutis laxa—the non-syndromic SVAS patients generally do *not* exhibit such features because of the different underlying mechanisms: elastin haploinsufficiency results in arterial stenosis, whereas an abnormal elastin protein leads to tissue laxity and vessel enlargement (31, 32). Therefore, the cardiovascular phenotypes resulting from ELN mutations can be broadly divided into two categories: one is the SVAS/PPS type, characterized mainly by elastic artery stenoses due to haploinsufficiency; the other is the aortic root dilatation/cutis laxa type, characterized by elastic fiber fragmentation leading to vessel dilatation and skin laxity.

### 4.3 Exon 28 frameshift mutation and its mechanism

In the case presented in our study, a novel frameshift mutation was identified in exon 28 of the ELN gene (c.1879_1883dup). This mutation is located near the carboxy terminus of elastin, close to the exon 29 region, placing it within the typical SVAS-associated mutation interval (exons 1–29) (29). The frameshift creates a premature termination codon, which would produce a truncated protein usually targeted for degradation via NMD. A similar case has been reported in a Finnish family of sextuplets with variant c.1983delG (p.Pro662Leufs*13 in exon 28) (11). All six siblings carrying this frameshift variant exhibited elastin arteriopathy of varying severity: some had only mild PAS, while others developed severe SVAS with coronary ostial stenosis leading to infantile death. This illustrates that an exon 28 frameshift mutation can likewise cause disease in a dominant manner, resulting in elastin deficiency and a predominantly SVAS/PAS phenotype (33, 34). It suggests that the mechanism for a mutation like p.Ala629LeufsTer15 (our case) is most likely haploinsufficiency: the mutant allele's product is degraded, leaving the patient with only half the normal elastin output (insufficient dosage from one allele). This haploinsufficiency mechanism aligns with the pathology of most non-syndromic SVAS cases. A reduced elastin level in the arterial wall leads to maldevelopment of the elastic lamina, which in turn causes arterial segments to become abnormally narrow and stiff.

It should be noted that certain variants near the exon 28 splice sites can result in different pathogenic mechanisms. For instance, Micale et al. reported seven novel ELN mutations in non-syndromic SVAS patients, which included frameshift changes leading to NMD as well as some mutations that exerted a dominant-negative effect (13). Interestingly, fibroblast experiments from one patient showed that the mutant allele produced a shortened, abnormal elastin peptide that became incorporated into elastic fibers and disrupted their assembly (a dominant-negative effect). This finding suggests that some special mutations—particularly those near the 3′ end of the gene that allow a portion of transcripts to escape NMD—can produce residual truncated proteins and trigger more complex effects beyond simple haploinsufficiency. However, for p.Ala629LeufsTer15 and most exon 28 mutations, the primary consideration remains an NMD-mediated elastin deficiency mechanism. These mutations ultimately lead to the same pathophysiological outcome as typical SVAS: a weakened elastic layer in large arteries and underdeveloped vessel lumens, culminating in localized stenoses (31).

In summary, exon 28 frameshift mutations have been documented in the literature, and their mode of pathogenicity is consistent with that of most ELN loss-of-function mutations—namely, elastin haploinsufficiency resulting in aortic and pulmonary artery stenoses. Patients with this class of mutation should undergo comprehensive cardiovascular evaluation, including imaging of the aorta, pulmonary arteries, and coronary arteries, with careful monitoring over time. Genetic counseling is also recommended, since these mutations are usually autosomal dominant; there may be asymptomatic carriers within the family who could be identified through cascade testing.

### 4.4 Clinical implications and follow-up management

In our proband, SVAS progressed from a peak gradient of 24 mmHg at 1 month to 35 mmHg at 9 months. Given the current mild-to-moderate gradient and absence of clinical symptoms, we plan continued conservative management with close pediatric cardiology follow-up and serial echocardiography to monitor gradients, left ventricular function, and associated vascular lesions. Cross-sectional imaging (CTA/MRI) and coronary ostial assessment will be considered if progression continues or prior to intervention. Surgical repair will be considered if the stenosis becomes hemodynamically significant (peak gradient ≥50 mmHg), if symptoms develop, or if there is evidence of ventricular dysfunction or myocardial ischemia.

## 5 Conclusion

Non-syndromic ELN gene mutations are a significant genetic cause of congenital SVAS and related arterial abnormalities. To date, over 100 non-syndromic ELN mutations have been reported, and these variants are closely linked to SVAS and other arterial anomalies. By synthesizing findings from these studies, clinicians can begin to predict aspects of a patient's clinical course based on the type of ELN mutation and its location. For example, one might anticipate the extent and severity of arterial stenoses, or recognize the likelihood of accompanying skin or pulmonary findings. Therefore, for patients suspected of having SVAS (after WBS has been ruled out), ELN gene testing should be performed to confirm the diagnosis. Once an ELN mutation is identified, we recommend a thorough cardiovascular assessment—including imaging of the aorta, pulmonary arteries, and coronary arteries—and regular follow-up to monitor the progression of any arterial stenoses. In addition, the patient and their family should receive genetic counseling and guidance on family planning.

As research into the mechanisms of ELN mutations and their modifiers progresses, we are hopeful that targeted interventions can be developed to address the arterial disease caused by elastin deficiency. Potential future approaches include gene therapy or modulation of molecular pathways, aiming to mitigate the effects of elastin haploinsufficiency and thereby improve patient outcomes. In summary, the pivotal role of non-syndromic ELN mutations in SVAS and other arterial abnormalities highlights that early diagnosis and individualized treatment are crucial for optimizing the clinical management of these patients.

## Data Availability

The original contributions presented in the study are included in the article/Supplementary Material. Further inquiries can be directed to the corresponding author.
