# Supplementary figures and images for: Case Report: Newly discovered ELN gene mutation in congenital heart disease: case analysis and review
Source: Front Pediatr. 2026 Jan 29;14:1671066. doi: 10.3389/fped.2026.1671066 (PMC12894336; doi:10.3389/fped.2026.1671066)

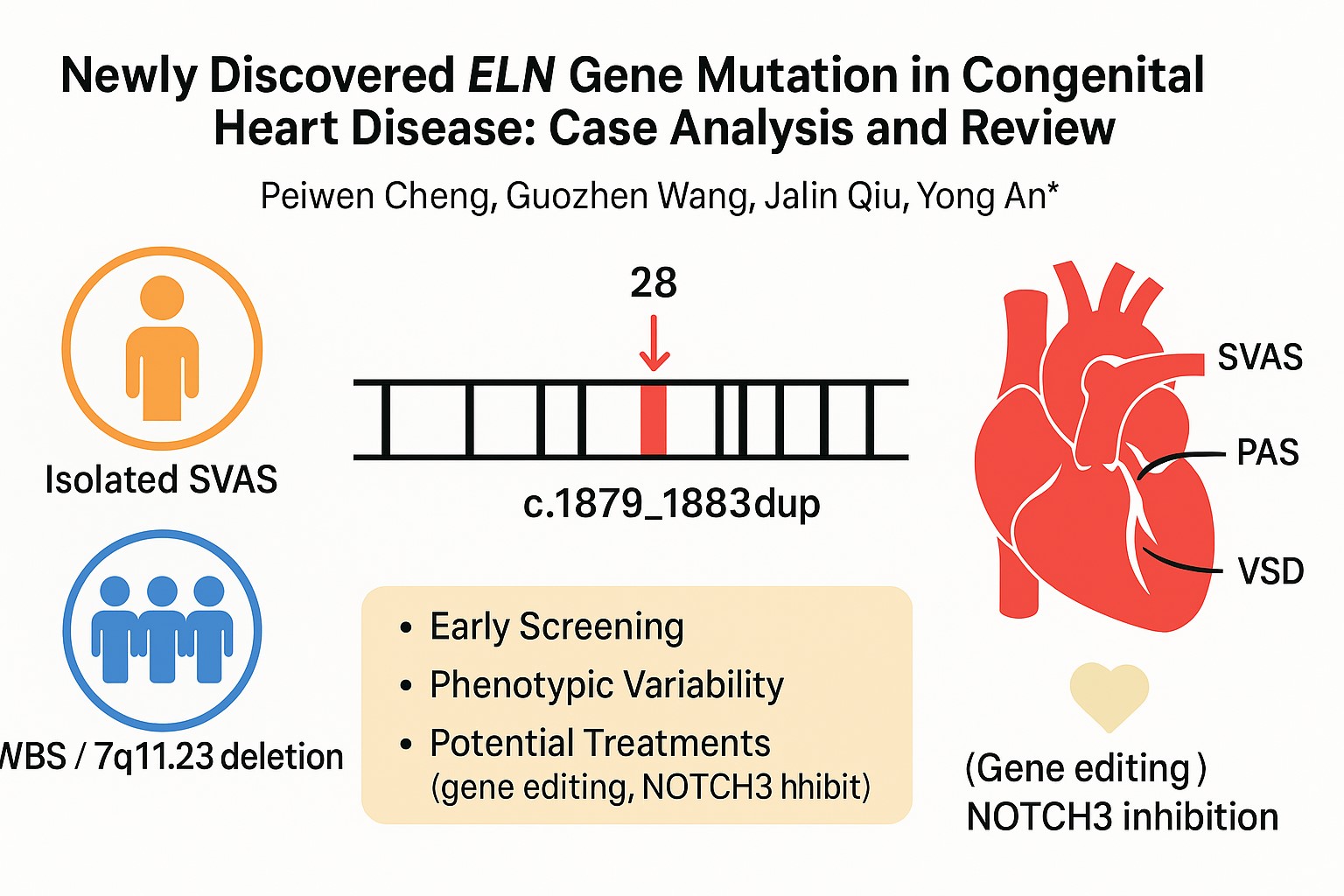

Supplement: Supplementary file 1 [file Image1.jpeg]
